# Emerging Trends in Deciphering the Pathogenesis of Human Diseases through Genetic Analysis

**DOI:** 10.3390/genes12010096

**Published:** 2021-01-13

**Authors:** David Q.-H. Wang

**Affiliations:** Department of Medicine and Genetics, Division of Gastroenterology and Liver Diseases, Marion Bessin Liver Research Center, Einstein-Mount Sinai Diabetes Research Center, Albert Einstein College of Medicine, Bronx, NY 10461, USA; david.wang@einsteinmed.org; Tel.: +1-(718)-430-8865

Any changes in gene expression or protein functions can cause abnormal anatomical, physiological, biochemical, and behavioral modifications in human beings, which can lead to disease. Curiosity about the pathogenesis of diseases has led to human beings using a variety of methods and tools to explore why and how such a process develops in the body. Many new methodologies for creating innovative medicine and providing novel insights into the mechanisms underlying disease pathogenesis have been obtained. One particular example is the rapid advance in the application of genetic technology from single-cell microbial systems to eukaryotic culture systems and up to multicellular whole-animal systems and the human body. As a result, the development of advanced genetic techniques has dramatically accelerated the growth of biotechnology and precise medicine and has deeply promoted our understanding of the pathophysiology of human diseases. To challenge the rapid development of these technologies, it is very important for researchers, scientists, and physicians to exchange ideas, share thoughts, and publish progress reports through various communication approaches. 

For this purpose, the Advanced Genetics Conference 2019 was the third edition of a Genomic Medicine meeting that was first started in 2017 and has since been held annually for researchers, scientists, and physicians around the world. The third edition of the conference was held on 13–15 November 2019 in Baltimore, MD, USA. This conference provided insights into the latest advances in the field of genetics, as well as opportunities to learn more about recently used genetic tools and techniques for exploring the pathophysiology of human diseases. In addition, the Advanced Genetics Conference 2019 provided a platform for sharing new ideas and thoughts with fellow researchers.

In this Special Issue of *Genes*, we acknowledge the contributions by several experts offering timely perspectives on emerging trends in deciphering the pathogenesis of human diseases through genetic analysis. This Special Issue includes nine publications addressing different topics on the genetic and epigenetic analysis of human diseases and the genomic regulation of cell functions at the gene level as examples illustrating various aspects of this field of discovery.

*Helicobacter pylori* (*H. pylori*) infection is a very prevalent digestive disease, affecting approximately 50% of the population worldwide. Some genetic variations could play a critical role in determining the susceptibility to this infection. BaniHani et al. studied the association between two single nucleotide polymorphisms (SNPs)—C1236T (rs1045642) and C3435T (rs1045642)—in the ATP-binding cassette, sub-family B, member 1 (*ABCB1*) gene, and the prevalence of *H. pylori* infection among Jordanians [1]. They found a significant association between C1236T and *H. pylori* infection, with a haplotype analysis of C1236T and C3435T SNPs showing that the TT haplotype was present in 22.7% of the positive cases compared to 30.7% of the negative controls. Therefore, there is an association between *ABCB1* SNPs and *H. pylori* infection in the Jordanian population.

African Americans display the highest prevalence rate of hypertension among American racial/ethnical groups, with over two-thirds of African American women being affected. To explore whether the differential gene expression of mRNAs and microRNAs (miRNAs) within peripheral blood mononuclear cells may contribute to this genetic difference in hypertension, Arkorful et al. investigated miRNA-1253 regulation of the Wiskott–Aldrich syndrome protein Verprolin-homologous protein 2 (WASF2, also known as WAVE2) and its relevance to racial health disparities through an in silico miRNA target prediction analysis [2]. Their results identified novel roles for miR-1253 and WASF2 in a hypertension-related disparities context. This may ultimately lead to the discovery of additional actin-related genes which are important in the vascular-related complications of hypertension and influence the disproportionate susceptibility to hypertension among African Americans in general and African American women in particular.

Although many RNA sequencing (RNA-seq) data have been deposited in public repositories, there are few open source tools with point-and-click interfaces that are versatile and offer a streamlined comprehensive analysis of RNA-seq datasets. To maximize the capitalization of these vast public resources and facilitate the analysis of RNA-seq data by researchers and scientists, Li et al. developed a new Web application called OneStopRNAseq for the one-stop analysis of RNA-seq data [3]. The pipeline has user-friendly interfaces and offers workflows for common types of RNA-seq data analyses, which can greatly facilitate the comprehensive and efficient analysis of private and public RNA-seq data.

It has been proposed that combining stem cell transplantation with therapeutic gene delivery via the genetic modulation of donor cells may be used for the treatment of ischemic cardiovascular disease. The Wnts are secreted glycoproteins in humans and Wnt modulation may offer a promising therapeutic strategy towards the restoration of myocardial tissues and an enhancement of cardiac functions following infarction. Indeed, the transduction of Wnt11 into mesenchymal stem cells (MSCs) (MSC^Wnt11^) promotes the differentiation of these cells into cardiac phenotypes. To study the paracrine effects of MSC^Wnt11^ on cardiac function and angiogenesis, Wang et al. investigated whether WNT11-conditioned medium could promote angiogenesis through activation of the non-canonical WNT-PKC-JNK signaling pathway [4]. They found that an injection of CdM^Wnt11^ into the peri-infarct region in an acute myocardial infarction model of rats significantly improves the cardiac function, reduces the infarct size, and increases the myocardial blood flow and blood vessel density in the ischemic area. Their results support the conclusion that Wnt11 released from MSC^Wnt11^ increases angiogenesis and improves the cardiac function via non-canonical Wnt-PKC-JNK-dependent pathways.

Although it is well-known that estradiol promotes the anorectic action of apolipoprotein A-IV (apoA-IV), the cellular and molecular mechanisms remain unclear. Liu et al. studied whether estradiol enhances the anorectic effect of apolipoprotein A-IV through the estrogen receptor α (ERα) and the phosphatidylinositol 3-kinase (PI3K) signaling pathway in the nucleus tractus solitarius [5]. They found that the PI3K/Akt pathway is significantly activated by estradiol and apoA-IV, respectively, in primary neuronal cells isolated from the rat embryonic brainstem. Moreover, estradiol and apoA-IV can synergistically activate the PI3K/Akt signaling pathway and estradiol’s regulatory role in apoA-IV’s anorectic action occurs through the ERα-PI3K pathway in the nucleus tractus solitarius. Therefore, maneuvering PI3K/Akt signaling activation in the nucleus tractus solitarius may provide a novel therapeutic approach for the prevention and treatment of obesity-related disorders in females.

Emerging evidence suggests that chromosome 8q24 could be an important region in the genetic susceptibility to prostate cancer. Barry et al. studied whether *MYC* DNA methylation at 8q24 (six CpG sites from exon 3 to the 3′ UTR) in prostate tumor is associated with tumor aggressiveness, according to Gleason score analysis [6]. Their results showed that *MYC* methylation is not associated with *MYC* expression but is inversely associated with the expression of prostate cancer-associated non-coding RNA 1 (*PRNCR1*) after multiple comparison adjustment, thereby suggesting that prostate tumor *MYC* exon 3 hypomethylation may be associated with an increased aggressiveness.

It has been found that the gut microbiota is very important for the host’s health because it plays a critical role in the degradation of non-digestible polysaccharides, i.e., through the fermentation of resistant starch, oligosaccharides, and inulin, thereby strengthening the gut integrity or shaping the intestinal epithelium, harvesting energy, protecting against pathogens, and regulating host immunity. Notably, the composition of gut microbiota is rather individual-specific and normally, depends on both the host’s genotype and environmental factors. In this review article [7], Di Ciaula et al. discuss gut microbiota, as influenced by interactions between the environment and genetic background, in patients with familial Mediterranean fever. The studies of the bacterial profile in the gut showed that dominant and minor phyla are present in the gastrointestinal tract, with the bacterial density gradually increasing in the oro-aboral direction. The crosstalk between bacteria and the host within the gut strongly contributes to the host’s metabolism, as well as to structural and protective functions. Dysbiosis can develop following aging, diseases, an inflammatory status, and antibiotic therapy. Growing evidence has clearly demonstrated a possible link between the microbiota and familial Mediterranean fever, through a shift of the relative abundance in microbial species. It remains to be further investigated to which extent such perturbations of the microbiota are relevant in driving the phenotypic manifestations of familial Mediterranean fever with respect to the genetic background.

Genome instability could increase tumor formation because the accumulation of genetic alterations ranging from single nucleotide mutations to chromosome rearrangements could promote cells towards malignancy. In the absence of any exogenous agent, a single human cell is subjected to about 70,000 DNA lesions each day. Therefore, some physiological cellular processes could contribute to DNA damage and induce DNA damage responses in the cell, which could be influenced by the three-dimensional chromatin architecture and epigenetic regulation. However, these can be altered during the malignant transformation of cells. In this review article [8], Mehrotra and Mittra discuss the origin of genome instability and determinants of the mutational landscape in cancer cells, with a special focus on how replication stress, oncogene activation, chromatin dynamics, and the illegitimate recombination of cell-free chromatin particles deregulate cellular processes in cancer cells and contribute to their evolution. Understanding these cellular and molecular mechanisms could depict the mutational landscape in cancer cells during different stages of tumorigenesis and explicate their impacts on the treatment strategies and prognosis.

After being activated by cholecystokinin (CCK), the CCK A receptor (CCKAR) can regulate gallbladder and small intestinal motility under normal physiological conditions. However, the *Cckar* gene has been identified to be an important gallstone gene—*Lith13*—in inbred mice through a powerful quantitative trait locus analysis. As summarized in the review article by Wang et al. [9], dysfunctional *CCKAR* can significantly increase the susceptibility to cholesterol gallstone formation in humans and mice through two primary mechanisms: Impairing gallbladder emptying and delaying small intestinal transit. The former causes gallbladder hypomotility, biliary sludge (the precursor of gallstones), and microlithiasis, and the latter promotes intestinal cholesterol absorption as a major source of the hepatic hypersecretion of biliary cholesterol, thus leading to cholesterol-supersaturated gallbladder bile. If these two defects could be restored or prevented by potent CCKAR agonists, the risk of developing cholesterol gallstones could be reduced dramatically.

In conclusion, genetic and epigenetic investigations on the pathogenesis and pathophysiology of human diseases have become even more complex and exciting when considering that the discovery of disease genes and their signaling or regulatory pathways, as well as their dysfunctions in the body, has paved the way to multidimensional, multidisciplinary, and translational studies at a cellular and molecular level. Furthermore, understanding whether and how disease genes and their signaling or regulatory pathways work is essential to deciphering the complex pathophysiological mechanisms underlying the processes and severity of human diseases. A new era in which disease genes and their signaling or regulatory pathways will represent potential therapeutic targets for several disorders is hopefully approaching in the near future.

Finally, we sincerely hope that this Special Issue of *Genes* will help both basic scientists and clinical investigators in their difficult daily tasks to expand their basic, translational, and clinical research on the pathogenesis of human diseases and explore novel therapeutic approaches to these diseases.

## References

[1] Banihani M.N., Khabour O.F., Alzoubi K.H., Bashir N.A., Shakhatreh M.A.K., Sabi S.H., Alrabadi N. (2020). The Association between *ABCB1* C1236T/C3435T SNPs and *H. pylori* Infection among Jordanians. Genes.

[2] Arkorful M.A., Hooten N.N., Zhang Y., Hewitt A.N., Sanchez L.B., Evans M.K., Dluzen D.F. (2020). MicroRNA-1253 Regulation of WASF2 (WAVE2) and its Relevance to Racial Health Disparities. Genes.

[3] Li R., Hu K., Liu H., Green M.R., Zhu L.J. (2020). OneStopRNAseq: A Web Application for Comprehensive and Efficient Analyses of RNA-Seq Data. Genes.

[4] Wang J., Gong M., Zuo S., Xu J., Paul C., Li H., Liu M., Wang Y.-G., Ashraf M., Xu M. (2020). WNT11-Conditioned Medium Promotes Angiogenesis through the Activation of Non-Canonical WNT-PKC-JNK Signaling Pathway. Genes.

[5] Liu M., Shen L., Xu M., Wang D.Q.-H., Tso P. (2020). Estradiol Enhances Anorectic Effect of Apolipoprotein A-IV through ERα-PI3K Pathway in the Nucleus Tractus Solitarius. Genes.

[6] Barry K.H., Mohanty K., Erickson P.A., Wang D., Shi J., Rose G., Cellini A., Clark K., Ambulos J.N., Yin J. (2020). *MYC* DNA Methylation in Prostate Tumor Tissue Is Associated with Gleason Score. Genes.

[7] Di Ciaula A., Stella A., Bonfrate L., Wang D.Q.-H., Portincasa P. (2020). Gut Microbiota between Environment and Genetic Background in Familial Mediterranean Fever (FMF). Genes.

[8] Mehrotra S., Mittra I. (2020). Origin of Genome Instability and Determinants of Mutational Landscape in Cancer Cells. Genes.

[9] Wang H.H., Portincasa P., Liu M., Tso P., Wang D.Q.-H. (2020). An Update on the Lithogenic Mechanisms of Cholecystokinin a Receptor (CCKAR), an Important Gallstone Gene for *Lith13*. Genes.

